# Advancing brain tumor epidemiology – multi-level integration and international collaboration: The 2018 Brain Tumor Epidemiology Consortium meeting report 

**DOI:** 10.5414/NP301148

**Published:** 2018-10-22

**Authors:** Kimberly J. Johnson, Helle Broholm, Michael E. Scheurer, Ching C. Lau, Johannes A. Hainfellner, Joseph Wiemels, Judith Schwartzbaum

**Affiliations:** 1 Brown School Master of Public Health Program, Washington University, St. Louis, MO, USA,; 2 Department of Pathology, Copenhagen University Hospital, Rigshospitalet, Denmark,; 3 Department of Pediatrics, Section of Hematology-Oncology, Baylor College of Medicine, Houston, TX,; 4 Division of Hematology-Oncology, Connecticut Children’s Medical Center, Hartford, and The Jackson Laboratory for Genomic Medicine, Farmington, CT, USA,; 5 Institute of Neurology, Medical University of Vienna, Austria,; 6 Department of Preventive Medicine, University of Southern California, Los Angeles, CA, and; 7 Division of Epidemiology, College of Public Health, Ohio State University, Columbus, OH, USA

**Keywords:** brain tumors, epidemiology, integration

## Abstract

The Brain Tumor Epidemiology Consortium (BTEC) is an international consortium that aims to foster multicenter and inter-disciplinary collaborations that focus on research related to the etiology, outcomes, and prevention of brain tumors. The 19^th^ annual BTEC meeting was held in Copenhagen, Denmark, on June 19 – 21, 2018. The meeting focused on forming international collaborations and integrating multiple data types for the next generation of studies in brain tumor epidemiology. The next BTEC meeting will be held in Southern California in June 2019.

## Introduction 

The Brain Tumor Epidemiology Consortium (BTEC) provides a scientific forum for brain tumor epidemiology researchers whose research is aimed at improving our understanding of the etiology, outcomes, and prevention of brain tumors. In pursuit of its mission, BTEC members mentor junior investigators and other investigators who are new to brain tumor epidemiologic research. BTEC was founded in 2003 following a meeting sponsored by the U.S. National Cancer Institute’s (NCI) Division of Cancer Epidemiology and Genetics (DCEG) and the U.S. National Institutes of Health’s (NIH) Office of Rare Diseases (ORD). Since then, BTEC has evolved to become a self-directed consortium whose members focus on the epidemiological aspects of brain tumors, including studies focused on identifying both environment and genetic risk factors, global patterns in brain tumor incidence and survival, and methodological work including methods for registry classification that lead to improved surveillance. BTEC is a U.S. National Cancer Institute-designated consortium and a non-profit 501(c)(3) corporation. 

**Figure 1.**
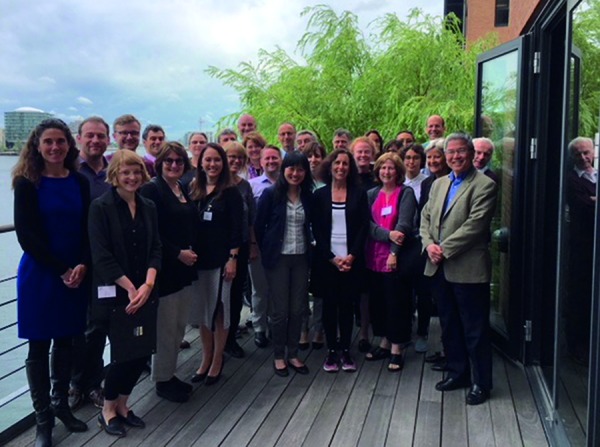
Attendees at the 2018 BTEC meeting in Copenhagen, Denmark.

BTEC held its 2018 annual meeting in Copenhagen, Denmark, with the theme *“Advancing Brain Tumor Epidemiology Multi-level Integration and International Collaboration”*. Understanding the factors associated with brain tumor etiology and outcomes requires integration of multiple data types (e.g., -omics, clinical, demographic, environmental), novel statistical approaches, and international collaboration due to the rarity of this tumor type. Speakers addressed the theme from these multiple perspectives at the meeting. The program committee included Board of Director members: Helle Broholm, PhD, Co-Presidents Ching Lau, MD, PhD, and Johannes Hainfellner, MD; Vice President Judith Schwartzbaum, PhD; Secretary Kim Johnson, MPH, PhD, Treasurer Michael Scheurer, PhD, MPH; and past President Joseph Wiemels, PhD. The meeting was coordinated by Ms. Bénédicte Clement of Montpellier, France. The meeting included four keynote addresses and a panel discussion with research relevant to the meeting theme. Additionally, there were 14 abstract presentations by junior and senior brain tumor researchers. Scientists from 10 countries representing a broad range of disciplines associated with brain tumor research attended the meeting. A summary of the scientific content of the meeting is provided in this report. A picture of attendees of the 2018 BTEC meeting is shown in Figure 1. 

## Summary of keynote lectures 

**Kjeld Schmiegelow, MD, of Rigshospitalet, Copenhagen, Denmark,** gave a talk entitled *“Brain tumors in childhood – Staging brain tumor epidemiology from fetal life to cancer diagnosis”*

Prof. Schmiegelow discussed the challenging group of pediatric CNS tumors, occurring with an incidence rate of ~ 40 per million per year, with the highest incidence in the world occurring in the Nordic countries. Initial symptoms are generally nonspecific and age-dependent, leading to delayed diagnosis. There is uncertainty about temporal trends and risk factors, and histologically and molecularly the tumors are very heterogenous and very different from CNS tumors in adults. The CNS tumors of children are the most sequelae-burdened childhood cancer group, demanding intensive multimodality treatment. Furthermore, public registries have recorded both increased incidence and mortality rates supporting a true rise in incidence. Therefore, the need for multimodality strategies to improve the knowledge of these tumors including the biological background, epidemiological history, molecular genetic analysis with sequencing of tumor and germline DNA is urgently needed in order to improve treatments and decrease treatment-related side effects for these children. Dr. Schmiegelow talked about established risk factors [1] and two new large prospective studies, STAGING (Sequencing of Tumor and Germline DNA – Implications and National Guidelines) and the 5C (Danish Collaborative Comprehensive Childhood CNS tumor Consortium) coordinating both interdisciplinary and national strategies including gene technology and molecular biology for pediatric brain tumors. These large initiatives will produce new findings, and we look forward to hearing about these results in the future. 

**Lily Li, MD, MS, of Mount Sinai School of Medicine, New York, NY, USA,** gave a talk entitled *“Integrating digital universe information towards precision medicine”* about the use of machine learning prediction methods within a highly detailed medical record database to tailor therapies for individuals with diabetes-related conditions. Dr. Li gave several examples of how digital information from various sources including electronic medical records, genetic marker data from biobanks and online catalogues can be used to finely phenotype patients into subtypes of diabetes for the development of more precise risk models and therapies that are finely tuned to the particular risk profiles of the patients. Dr. Li ended with a powerful example of what the next-generation health clinic might look like with wide adoption of machine learning to precisely characterize individual patient risks. 

**Devin Koestler, PhD, of the University of Kansas Medical Center, Kansas City, KS, USA** gave a talk entitled *“Immunomethylomics as a tool for understanding cancer risk and prognosis: a biostatistician’s perspective”*. Glioma is characterized by local and systemic immunosuppression thus making an understanding of the immune system in these patients essential. One aspect of the peripheral immune system, the leukocyte profile, may be used as a glioma biomarker and indictor of prognosis. DNA methylation, one of several epigenetic mechanisms, consists of the addition of methyl groups to cytosines, which occur adjacent to guanine nucleotides. This process is part of a normal developmental program unique in each tissue type and can vary in response to a physiologic or environmental signal and may result in altered gene expression. Immunomethylomics, a field recently pioneered by Koestler et al. [2], uses DNA methylation patterns to characterize peripheral leukocyte subtype distributions. Unlike previous methods of estimating leukocyte distributions, which require intact cells, immunomethylomics may be used on archived blood samples, thus making an important contribution to the analysis of archived samples in cohort studies. Once the leukocyte profile has been estimated, the methylation-derived neutrophil-to-lymphocyte ratio (mdNLR) appears to be a good indicator of inflammation in cancer patients and has been shown to associate with poor prognosis across several solid tumor types [2]. To determine whether the elevated mdNLR is associated with carcinogenesis or indicates tumor development, Koestler et al. [2] assessed the relationship between mdNLR estimated in pre-diagnostic blood, and lung cancer risk in a nested case-control study of individuals at high risk for lung cancer due to heavy smoking or substantial occupational asbestos exposure. They found evidence of significantly elevated mdNLR among future non-small cell lung cancer cases [3]. Wiencke, Koestler et al. [4] have additionally applied immunomethylomic profiling to glioma patients in the midst of therapy, and found that the mdNLR is predictive for survival. Thus the mdNLR may be used as an indicator of tumor development and treatment response. Further developments of this strategy will include the informatic delineation of additional relevant subtypes such as myeloid suppressive monocytes and granulocytes, and activating and suppressive lymphoid cells. 

**Dan Gatti, PhD, of The Jackson Lab in Bar Harbor, ME, USA,** gave a talk entitled *“Precision genetic mapping in diversity of outbred mice”*. Historically, experiments conducted with laboratory mice have predominantly used inbred mice strains, which are bred to be genetically identical and minimize experimental variability in phenotypes stemming from genetic differences between mice. However, phenotypes vary widely between strains and this has become recognized as a serious limitation of research using mice due to lack or reproducibility of study results using different strains and questions about whether the results can be extrapolated to humans [5]. Given these challenges, a pressing current question is what is the most appropriate preclinical mouse model? One active area of research is whether genetically diverse mice might provide more relevant preclinical results. To address this problem, diversity outbred (DO) mice were developed from 8 inbred founders at the Jackson Lab. The DO genomes are mosaics of the 8 founders. These mice have been used for projects such as mapping genes that influence chemotherapy-induced hematotoxicity [6] and identifying germline modifiers of metastasis in human prostate cancer [7]. For brain tumor research specifically, Dr. Gatti noted that these mice might be useful for studies trying to understand glioma initiation, treatment of different types of glioma, biomarkers of glioma, and interacting genes that influence glioma penetrance and severity. 

## Symposium on data integration: The promise and success of international collaborations 

**Melissa Bondy, PhD,** **of Baylor College of Medicine, Houston, TX, USA,** gave a talk outlining the successes of the Gliogene Consortium, which initiated the first collaborative research projects from members of BTEC, and included 14 sites across the United States and Europe. The Gliogene Family Study focused on unraveling the genetic susceptibility to familial glioma. They identified several novel mutations, including the gene, POT1, associated with familial glioma. The family study is continuing at Baylor College of Medicine and they are recruiting new families to follow up on these findings. Shortly after the Gliogene study was initiated, the Consortium began the Glioma International Case-Control Study. This large study has identified, to date, 13 new risk loci from a genome-wide association analysis, and is now focused on resolving previously suggested environmental risk factors (e.g., history of allergies/asthma, chicken pox, or seizures) and identifying novel gene-environment interactions for glioma risk. In addition to describing the research being conducted in the Consortium, Dr. Bondy also discussed the availability of data and samples from the Consortium for other collaborative research projects. 

**Jennifer Harris, PhD, of the Norwegian Institute of Public Health in Oslo, Norway,** gave a talk entitled *“Can you bank on it? International collaborations in biobanking studies.”* She provided a summary of the “lessons learned” in her experience with large international collaborations. In particular, she discussed the practical challenges in the data ecosystem for these large-scale studies. One of the primary issues is with data integration, a major theme for the meeting. This included knowing what data are available, dealing with a wide variety of data types, and collaborating across countries, and data harmonization. Data governance also poses a challenge, different regulatory frameworks cover different sets of data, and it is important that all contributors are able to send and receive data and specimens related to the studies being conducted. Reusability was another key consideration for developing a data ecosystem that permits the data to be used beyond the time frame of a single project. She also emphasized that many initiatives have developed openly available tools that new projects may find useful to facilitate their own work with data harmonization and data sharing. Finally, she also spoke about the need to engage participants in determining scientific priorities, as much as possible and feasible. 

**Jill Barnholtz-Sloan, PhD, MS, of Case Western Reserve University in Cleveland, OH, USA,** shared her experiences in setting up the Ohio Brain Tumor Study, a statewide biorepository network to facilitate studies of brain tumor genetics. She described the multiple collaborative projects where the Ohio Brain Tumor Study was leveraged to assess risk factors, prognostic factors and molecular features for diagnosis for brain tumors, including The Cancer Genome Atlas project. She also described how their participation in The Cancer Genome Atlas directly resulted in changes for molecular diagnostics for brain tumors in the form of revised World Health Organization International Classification of Disease Oncology coding which has now been implemented worldwide. She further described the challenges with setting up the informatics infrastructure to support the project across the multiple medical record systems at the partner sites, with a particular focus on the informatics infrastructure being set up in Cleveland, OH. 

## Abstract presentations 

There were 14 abstracts presented over two days that covered topics including descriptive epidemiology of brain tumors, risk factors, access to care, and statistical methodology pertaining to brain tumor research. The first abstract session began with four presentations. The first presentation entitled *“Longer genotypically estimated leukocyte telomere length is associated with increased meningioma risk in females, but not with clinical outcomes”* was given by **Ivo S. Muskens, MD,** of the Department of Neurosurgery at Brigham and Woman’s Hospital in Boston, MA, USA. Dr. Muskens presented the results from a case-control study of 1,053 meningioma patients and 4,437 European descent controls. Using the estimated leukocyte telomere length (LTL) based on single nucleotide polymorphisms (SNPs) previously associated with longer telomere lengths. The major finding of this study was that longer genotypically estimated LTL was associated with an increased risk of meningioma development after correction for gender and ancestry, but not progression. The second presentation entitled *“Increasing incidence of central nervous system (CNS) tumors (2000 – 2012): findings from the Gironde Registry of CNS tumors (France)”* was given by **Camille Pouchieu, PhD,** of the University of Bordeaux in France. The aims of this study were to describe trends in CNS tumor incidence overall and by sex, age group, and histologic subtype. There were 3,515 CNS tumors diagnosed during the study period. The incidence of CNS tumors significantly increased over the study period by 2.7% (p < 0.0001), which was more pronounced in women than men and evident in all age groups. The trend was especially pronounced for meningiomas. 

The third presentation entitled *“ABTR-SANO Pattern of care study glioblastoma – update”* was given by **Andreas Hainfellner** of the Medical University of Vienna in Austria. Dr. Hainfellner gave an update on a study that he presented at the BTEC conference in 2016 [8]. In the current population-based study, characteristics of 571 patients, treatment patterns, and survival from GBM were examined in patients diagnosed between 1/2014 and 12/2015. Follow-up was through 12/2017. Total or subtotal resections occurred in a majority of patients (67.4%), and most patients (86.3%) started on a combined treatment schedule. Median survival was 13 months in the cohort and differed by patient characteristics including age, extent of resection, and MGMT status. The conclusion from this study was that the current standard of care is in wide practice in neuro-oncology units in Austria. The fourth presentation in the first session entitled *“Leveraging genomic data to identify risk factors for childhood ependymoma”* was given by **Kyle Walsh, PhD,** of the Duke University Medical Center in Durham, NC, USA. A case-control study including 360 individuals diagnosed with pediatric ependymomas at < 20 years from the California Cancer Registry prior to 2010 were identified. Cases were linked to their archived newborn blood spots collected from 1982 to 2009 and the CA birth registry. Two controls per case were identified from the CA birth registry along with their archived newborn blood spots for comparison. SNPs previously identified in genome wide association studies were genotyped and polygenic scores for anthropometric traits, leukocyte telomere length, and immune cell profiles were constructed. The main conclusions from this study were that children who were genetically predisposed to be taller, have longer telomere length, and decreased circulating myeloid cell counts may be at increased risk of ependymoma. 

The second abstract session began with two junior investigator award presentations that were sponsored by the American Brain Tumor Association. The first junior investigator award presentation was given by **Jeremy Schraw, PhD,** of the Baylor College of Medicine in Houston, TX, USA. In his talk entitled *“The risk of brain tumors in children and adolescents with congenital anomalies: a population-based assessment among > 10 million live births”*, he reported the results from a study that linked birth certificates to birth defects and cancer registries in four U.S. states (Texas, Michigan, North Carolina, and Arkansas). Based on almost 300 individuals with both a brain tumor and a non-syndromic congenital anomaly diagnosis, the major findings from this study were a three-fold increased hazard of childhood brain tumors (CBTs) in children with non-syndromic anomalies and a six-fold increased risk of CBTs in children with central nervous system anomalies. 

The second junior investigator award presentation was given by **Quinn Ostrom, PhD,** also of the Baylor College of Medicine in Houston, TX, USA. In her talk entitled *“Evaluating glioma risk associated with extent of European admixture in African-Americans and Latinos”*, she described results from a study that aimed to assess whether excess European Ancestry at established glioma risk loci was associated with glioma risk in individuals with non-European ancestry. Using data from over 500 individuals with > 40% African Ancestry and > 15% Native American Ancestry in the Glioma International Case-Control Study and GliomaSE Case-Control Study, the main findings from Dr. Ostrom’s study were that glioma risk in African Americans and Hispanics may be associated with an excess of local European ancestry variants at glioma risk loci previously identified in European populations. 

Following the junior investigator award talks were four presentations. **Kim Johnson, MPH, PhD,** of Washington University in St. Louis, St. Louis, MO, USA, gave a talk entitled *“The effects of insurance status on childhood brain tumor survival in the United States”*. Dr. Johnson reported results from a cohort study using data from the U.S. National Cancer Database. During the study period from 2004 to 2009, 8,025 individuals diagnosed with pediatric brain tumors < 15 years were included in the dataset. The main finding from the study was inferior survival for both Medicaid-insured (11% higher hazard of death) and uninsured children (26% higher hazard of death) vs. privately insured children at diagnosis. Next a talk entitled *“A novel risk model to define the relative benefit of maximal extent of resection within prognostic groups in newly diagnosed glioblastoma”* was given by **Annette M. Molinaro, PhD,** of the University of California San Francisco, San Francisco, CA, USA. In the first study of its kind, Dr. Molinaro reported preliminary results from a methodological study with the goal of developing a new roadmap for cytoreductive surgery. The study combined data on volumetric extent of resection with molecular and clinical factors and examined different combinations of factors on survival using the Partitioning Deletion/Substitution/Addition algorithm (https://cran.r-project.org/web/packages/partDSA/vignettes/survival.pdf). The ultimate goal of this research is to develop a classifier that can be used to inform future surgical strategies for glioma patients according to both tumor and clinical characteristics. 

Following Dr. Molinaro’s talk, **Baran Atli,** of the Medical University of Vienna, Vienna, Austria, gave a talk on *“Descriptive population-based epidemiology of intracranial germ cell tumors in Austria”*. The aims of this study were to estimate incidence and survival rates of CNS germ cell tumors (GCTs) in Austria and to investigate differences by sex and age, anatomic types, and histological subtypes. Data on incidence and survival from Austria the U.S. and Japan were obtained from the Austrian Brain Tumor Registry (ABTR) from 2005 to 2011, Statistics Austria, the U.S. National Cancer Database (1990 – 2003), the Brain Tumor Registry of Japan (1984 – 2000), the Surveillance, Epidemiology, and End Results Program (2004 – 2008), and the Japan Surveillance Research Group (2004 – 2006). Over the study period of 2005 – 2011, 31 cases were diagnosed with CNS GCTs for an incidence of 0.8/1,000,000. Five-year survival was 96%. Although Austrian incidence rates were comparable to Japan and the United States, survival was higher (96% vs. 75%). In the final talk of this session, **John L. Villano, MD, PhD,** of the University of Kentucky, Lexington, KY, USA, gave a talk on *“Brain tumor and prognostic estimates: Results from the National Cancer Database”*. Dr. Villano reported the results of a comprehensive analysis of the largest source of hospital-based cancer patient information in the world, to examine patterns of care and survival in patients treated in U.S. comprehensive cancer centers. Among 464,852 patients with brain tumors from 2004 to 2014, one of the major findings was the high percentage of patients that did not receive any form of treatment as initial therapy, along with the small number of reported molecular profiling information, and the weight of socioeconomic factors in treatment decisions. Dr. Villano concluded that despite some limitations of the NCDB data – including lack of specific chemotherapy agent used, and inability to link with other data sources – it is a useful data source for evaluating patterns of care, information that is not available in other U.S. data sources. 

In the final abstract session on the second day of the conference, four presentations were given. **Angela Zumel-Marne,** predoctoral researcher of the Institute for Global Health of Barcelona in Barcelona, Spain, gave a talk entitled *“Clinical Characteristics of brain tumors in young people: results of the international Mobi-Kids study”*. Miss Zumel-Marne reported results from the Mobi-Kids study that included 899 brain tumor cases aged 10 – 24 years at diagnosis from 14 countries. Miss Zumel-Marne reported data on the frequency of morphologies, topographies, timing before diagnosis and presenting symptoms in Mobi-Kids cases and found that the symptoms depend on location and type of tumor, among other results. Miss Zumel-Marne’s talk was followed by a talk from **Luc Bauchet, MD**, of the University Montpellier Hospital, in Montpellier, France. His talk was entitled *“Adolescent and young adult primary central nervous system tumors diagnosed in France: preliminary results”*. Dr. Bauchet reported the results from a study of the French Brain Tumor Database that included 50,510 cases on newly diagnosed and histologically confirmed PCNST from 2008 to 2012. The major findings from this study were that French and U.S. data were very similar. Only one important difference was noted that the incidence of the pituitary tumors was much lower in the French data. The French Brain Tumor Database records brain tumors with histological diagnosis only, and an important number of pituitary tumor patients do not have surgery. Secondly, the quasi totality of adolescent and young adult (AYA) patients with malignant brain tumor had surgery with histological analysis. Thirdly, for countries where brain tumor registration is not mandatory, a national histological registry is a good way to estimate epidemiology and study medical care for AYA patients with malignant brain tumors. The final two presentations of the meeting continued to reflect the wide variation in expertise that the BTEC meetings have traditionally encompassed. **David J. Cote** of Brigham and Women’s Hospital and Harvard Medical School Boston, MA, USA, spoke about *“Body mass index, waist circumference, height and body somatotypes and risk of glioma and glioblastoma”*. Using data from the Nurse’s Health Study (NHS) that included 121,696 women and the Health Professionals Follow-up Study (HPFS) that included 51,400 men, a total of 508 cases of incident glioma were diagnosed during the follow-up periods. Major findings from this study were that adult body mass index (BMI) and waist circumference were not associated with glioma. However, young adult BMI was associated with an increased risk as was greater height with a more pronounced effect in women. The concluding presentation by **Christian T. Stackhouse,** of the University of Alabama at Birmingham, Birmingham, AL, USA, was entitled *“Exploring the role of lncRNA in radiation resistances in a GBM-PDX model of tumor recurrence”*. Employing PDX models, Mr. Stackhouse presented the aims for a study that has the goal of developing PDX models to assess mechanisms of inherited and acquired therapy resistance in GBMs that invariably reoccur 6 – 9 months post-surgical resection. In particular, the study will explore the involvement of long non-coding RNAs in resistance. 

## Conclusion 

The 2018 BTEC meeting covered a wide-array of topics in both adult and childhood brain tumors, with a focus on the power of multi-level data integration and international collaboration. The conference key note speakers addressed topics on research aspects not traditionally associated with brain tumor epidemiology but whose techniques and concepts can help move the field forward. For instance, the 2018 meeting featured a presentation on an animal model of genetic susceptibility (the diversity outbred mouse model, D. Gatti) which may help develop the burgeoning field of glioma genetics, long guided by BTEC collaborative investigations in humans. The increasing evidence for immune modifiers in glioma will need incorporation of innovative analytical techniques which can utilize typical population-based specimens such as frozen blood, which may involve immunomethylomics (D. Koestler). Furthermore, the high dimension omics-rich data of current genetic, epigenetic, and traditional epidemiologic datasets of BTEC and others will require higher level data integrative methods such as machine learning especially if we want to integrate them with clinical data extracted from electronic medical records (L. Li). These new techniques will require new and larger patient cohorts that seek to follow brain cancers from nascent formation through to the end of therapy to address the full course of disease (K. Schmeigelow). Finally, all of these research methods require larger studies which can pool and replicate findings to ensure generalizability and speed discoveries to new prevention and treatment modalities (M. Bondy, J. Harris, J. Barnholtz-Sloan). All of this work will indeed require the recruitment of new energy and talent to brain cancer research, as exemplified by the diverse abstract submissions to this meeting. Brain cancer incidence has not decreased over the last half century of the modern epidemiology era, demanding continued attention to this disease and productive collaboration on finding its causes. 

## Acknowledgment 

We are thankful for the generous meeting support of the American Brain Tumor Association, the Brain Tumor Foundation of Canada, Jackson Labs, and NanoString. We are also thankful to the tireless dedication of Bénédicte Clement who helps us coordinate our annual meetings. 

## Conflict of interest 

All authors declare that there are no conflicts of interest.
